# Quality assessment and its impact on clinical performance of a biosimilar erythropoietin: A simulated case study

**DOI:** 10.1016/j.biologicals.2019.10.006

**Published:** 2019-11

**Authors:** Robin Thorpe, Gustavo Grampp, Hye-Na Kang, Ivana Knezevic

**Affiliations:** a Welwyn, United Kingdom; b Biotherapeutics Group of the International Federation of Pharmaceutical Manufacturers and Associations (IFPMA), United Kingdom; c World Health Organization, Department of Essential Medicines and Health Products, Avenue Appia 20, CH-1211, Geneva, Switzerland

**Keywords:** Biosimilar, Similar biotherapeutic product, Erythropoietin, Regulatory evaluation, Case study, WHO

## Abstract

The case study described in this paper was developed for the purpose of training for a better understanding of principles relating especially to a comprehensive evaluation of multiple quality attributes as outlined in the WHO *guidelines on evaluation of similar biotherapeutic products*. It is also to emphasize the importance of an understanding of the critical quality attributes and a risk assessment of the impact on clinical performance. It was prepared to mimic a real situation in which regulators need to evaluate the differences in quality attributes known to have potential impact on clinical activity. Erythropoietin has been identified as one of the important glycosylated therapeutic proteins and a good example to illustrate how structural characteristics would affect product efficacy and safety. The case study illustrates biosimilarity assessment of a candidate of erythropoietin biosimilar and the important quality attributes that need to be considered in order to understand the importance of structure-function relationships as they contribute to the stepwise evaluation of biosimilarity. This paper reflects the outcomes of the case study exercise and discussion from two WHO implementation workshops held in Ghana (September 2015) and Denmark (July 2017).

## 1 Introduction

Since the World Health Organization (WHO) *guidelines on evaluation of similar biotherapeutic products* (SBPs) were adopted by the WHO Expert Committee on Biological Standardization (ECBS) in 2009 [1], WHO has organized 8 workshops to facilitate the implementation of the evaluation principles of the WHO guidelines into regulatory practices in several countries. One of the main outcomes of various implementation workshops was the agreement that SBPs should not be regulated like small molecule generics and a head to head comparability exercise of quality, safety and efficacy is essential for a product to be considered a SBP. The comparability exercise includes in depth analytical comparisons of structural and functional attributes, followed by comparative nonclinical studies (where appropriate), and clinical pharmacology and immunogenicity studies. Additional studies may be required to address any residual uncertainties from the comparability exercise. If major differences are found in the comparability exercise, the product cannot be called ‘similar’. However, the regulatory framework for the licensing of SBPs permits some analytical differences between the SBP and the reference biotherapeutic product (RBP) [1]. Such differences should be assessed for their potential impact on clinical safety and efficacy of the SBP and justification (for example, using the producers study results or published data) for allowing such differences should be provided. This latter information must show that any observed differences have no significant impact on clinical safety and efficacy. Increased knowledge of the relationship between product quality attributes and clinical outcomes of originator products (i.e. RBPs) facilitates development of SBPs. Analytical similarity assessment involves identification of “all clinically relevant quality attributes, i.e. product attributes that may impact clinical performance” [1].

WHO has developed several fictional case studies for monoclonal antibody products as SBPs mimicking a real situation of regulatory evaluation of SBPs [2,3]. This case study on erythropoietin (EPO) was intentionally developed for the purpose of group work practice at WHO implementation workshops to highlight important aspects of biosimilarity evaluation, in particular evaluation of quality attributes and the importance of understanding structure-functional relationships [4], as they contribute to the stepwise evaluation of biosimilarity as outlined in section 8 of the guidelines [1]. EPO has been identified as one of the important glycosylated therapeutic proteins and a good example to illustrate how structural characteristics (e.g. glycosylation and product or process related impurities) would affect product efficacy and safety (e.g. product half-life, immunogenicity).

## 2 Background information regarding the EPO products

EPO is the primary, and probably the sole mediator of hypoxic induction of erythropoiesis. It serves to: 1) maintain erythropoiesis under steady-state conditions (i.e. to keep RBC mass and haemoglobin concentrations (Hb) constant day by day, and 2) accelerate the recovery of RBC mass after haemorrhage. Erythropoiesis is a slow-acting process. It takes 3–4 days to detect the increase of the number of circulating red blood cells after a rise of EPO levels in plasma. The action of EPO on the erythropoiesis is augmented by other hormones namely testosterone, somatotropin and insulin-like growth factor 1. Endogenous EPO is a glycoprotein hormone that is mainly produced in the kidney. Kidneys secrete EPO under control of an oxygen sensing pathway that ultimately regulates the level of red blood cells in the circulation. Secreted EPO binds to the receptors of red blood cell precursors in the bone marrow increasing the red blood cell count.

The availability of rDNA technology has allowed the production of a recombinant version of EPO (EPO product, epoetin) to treat patients who are deficient in EPO. The dose of EPO products should be closely titrated to achieve and maintain a required level of response, usually haemoglobin concentrations in individual patients.

EPO contains highly sialylated glycans that are essential for its pharmacology [5,6]. Glycosylation may differ between batches, or between EPO products, and this should therefore be monitored by defining and measuring glycoprotein critical quality attributes (CQAs).

The sialic acid content of EPO is important as it significantly affects half-life. Fully sialylated EPO has a relatively long half-life, whereas asialo EPO has a very short half-life; partially sialylated product has a half-life roughly proportional to the degree of sialylation. Because half-life is important for the clinical efficacy of EPO, manufacturers adopt manufacturing processes and purification processes to maximize sialylation. In this study, both reference product and biosimilar SBP1 are fully sialylated and asialo EPO content is negligible.

Recombinant EPO has an identical amino acid sequence to the naturally occurring hormone. Therefore, antidrug antibodies (ADAs) elicited via immunogenicity of an administered EPO therapy are likely to cross-react with endogenous EPO. In severe cases these ADAs lead to clinically relevant autoimmunity manifesting as pure red cell aplasia (PRCA) [7]. The cases of PRCA from one of the licensed EPO products (Eprex) showed that thorough understanding of the manufacturing and handling process is essential to defining the product characteristics and ultimately its safety and efficacy [4]. The reason for the increase in PRCA observed with Eprex is still not fully understood, but it has been agreed that some (rare) safety issues become apparent only in the post-marketing setting when larger numbers of patients are being treated [4].

## 3 Description of the case study

### 3.1 Strategy

As stated in the WHO guidelines [8], quality assessment of biotherapeutic products (BTPs) requires the use of a panel of complementary methods which differ in their underlying scientific/technical basis to provide an overall picture of the quality of the product. This applies to SBPs but with an added proviso that a comparability assessment is also required which compares the quality of the SBP with the RBP; again a panel of orthogonal procedures is needed for this.

WHO defined CQA as “a physical, chemical, biological or microbiological property or characteristic that is selected for its ability to help indicate the consistent quality of the product within an appropriate limit, range or distribution to ensure the desired product quality” [8]. As the definition implies, the identification of CQA requires “knowledge of the relationship between product quality attributes and clinical activity of the RBP and related products, the clinical history of the RBP, and lot-to-lot differences for commercial lots of the RBP. For example, quality attributes such as composition and profile of glycosylation, biological activity that is known to be related to clinical activity, and receptor binding activity should be justified” [1]. A published case study describing a comparability exercise for a proposed EPO product manufacturing also illustrates the importance of understanding the structure-function relationships for glycosylation related critical quality attributes [9].

The critical attributes of EPO related to immunogenicity potential are not well understood, but it is generally held that higher order aggregates and particulates can increase risks of immunogenicity of BTPs [7].

Table 1 shows some methods which are of value for assessing EPO products. Criticality is an indication of the power of the assessment to provide information relevant for the safe and efficacious use of EPO and also its relative usefulness for quality evaluation. Many other methods could be employed, but their usefulness for quality assessment of EPO is generally low value.

**Table 1:**
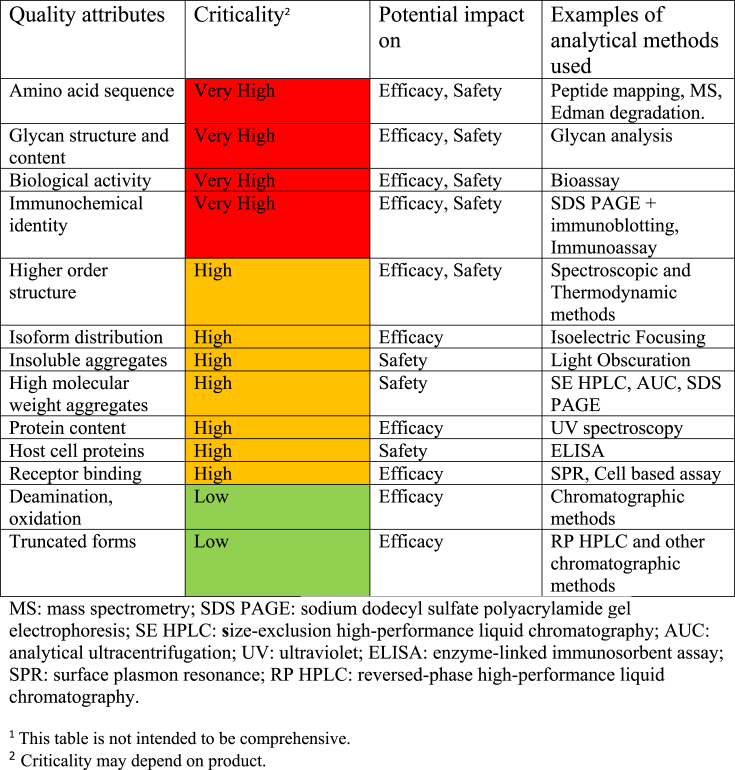
Criticality of quality attributes and their impact on clinical parameters of rDNA derived EPO products.^1^

For the biosimilar case study, two EPO products were compared. These were the RBP which was Eprex (International Nonproprietary Name: epoietin alfa) and a candidate SBP designated SBP1. Both were prepared using rDNA technology using Chinese Hamster Ovary (CHO) cells for expression and purified using a sequential series of established procedures. The clinical indications claimed for SBP1 were the same as for the RBP. A similar number of batches of the RBP and SBP1 were compared. The comparative analysis was carried out in the same laboratories on a head to head basis. The comparability exercise included head to head analytical studies, toxicokinetic studies in rats, clinical pharmacology studies in healthy volunteers, and a clinical efficacy/safety study in patients. The case study materials were intended to focus on the first stage of this stepwise exercise in order to focus the discussion on the implications of the analytical comparisons and the questions to be addressed in the subsequent non-clinical and clinical studies.

### 3.2 Methods used for testing selected quality attributes

**Fig. 1:**
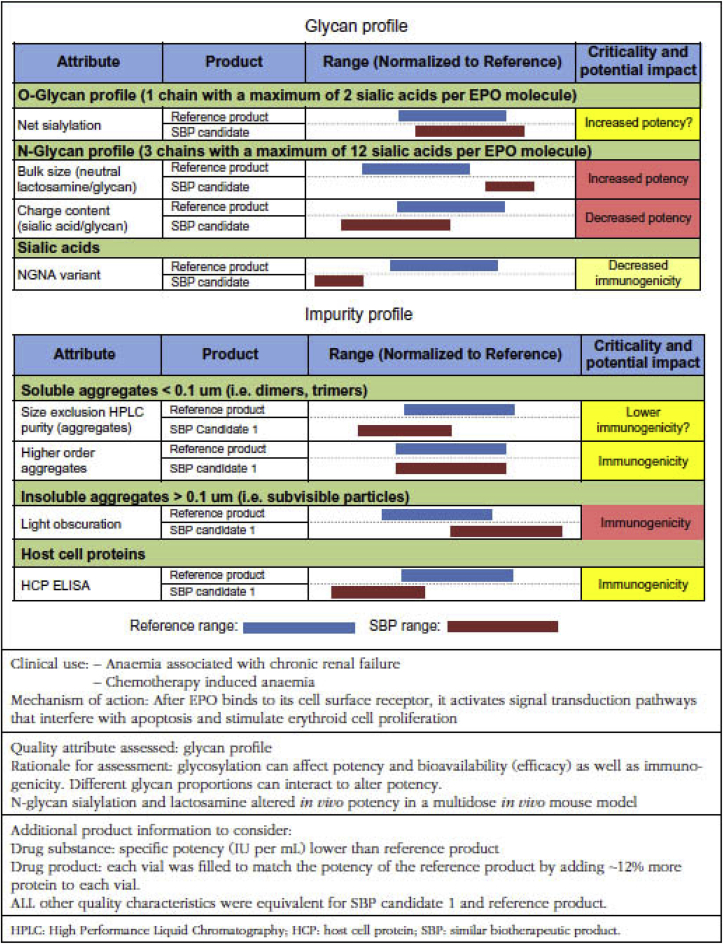
The comparison of selected subset of attributes with potential relevance. Reprint with permission. Walson PD, Thorpe R. First MENA educational workshop on regulation and approval of similar biotherapeutic products/biosimilars, Dubai, UAE, 1 September 2015. Generics and Biosimilars Initiative Journal (GaBI Journal). 2015;4(4):173-7 [10]

The methods included in Table 1 were used to characterize the two products and for head to head comparison. For this, the RBP and SBP1 were initially compared using an in vivo bioassay (normocythaemic mouse assay), in vitro receptor binding assays and a rat toxicokinetic study; the latter allows an approximate assessment of the increase in haemoglobin levels due to EPO treatment. The comparison also included peptide mapping and mass spectrometry (MS) techniques to provide amino acid sequence and glycan sequencing methods for carbohydrate analysis. Higher molecular weight impurities were assessed using physicochemical methods. Higher order structure (secondary and tertiary structure) was studied using a variety of spectroscopic and thermodynamic methods including near UV CD, differential calorimetry and Fourier transform infrared spectroscopy. These latter studies were conducted on EPO active substance isolated from the RPB and SBP1 using identical procedures and under the same formulation conditions. The methods were regarded as standardized (using suitable International Standards and reference materials) and validated to appropriate requirements as outlined in the WHO guidelines [8]. Accumulated data were analysed using relevant statistical methods and expressed as results for individual batches from the raw individual batch data in Fig. 1.

### 3.3 Results from the comparability study

The bioassay and receptor binding assays showed that the RBP and SBP1 showed very similar biological activity and receptor binding affinity when the limitations (variations) of the assays are taken into account. Similarly, MS and peptide mapping demonstrated identical amino acid sequence for the two products, which was also identical to the known natural human EPO sequence. Spectroscopic and thermodynamic techniques show equivalent secondary and tertiary (higher order) structures for the RPB and SBP1 within the limits of the methods. The rat toxicokinetic study, although limited by development of neutralizing antibodies in animals after the repeat doses of EPO required, revealed similarity of both the RBP and SBP1in their abilities to increase haemoglobin levels in treated rats.

For the purposes of the case study, the data to be specifically considered by the participants was limited to those shown in Fig. 1. This figure compares attributes for the RBP and SBP1 derived from data produced using appropriate methodologies (see Table 1 and appropriate text in section 3.2 above). The data has been summarised as a range for each product which includes the individual results for all the batches analysed. The information included in Fig. 1 and the supporting data presented in the 1st paragraph of this section above should be assessed by case study participants to provide an assessment of the potential of SBP1to be entered into a clinical trial as a biosimilar EPO product and also to answer the questions posed in section 4 below.

## 4 Methodology of the case study practices

This case study was prepared for WHO implementation workshops and has been used in two workshops. There were 27 regulators from 16 countries (i.e. Algeria, Botswana, Burkina Faso, Burundi, Ethiopia, Gambia, Ghana, Kenya, Mozambique, Nigeria, Sierra Leone, South Africa, Tanzania, Uganda, Zambia, Zimbabwe) in the workshop for English speaking countries in Africa Region [4], and 30 regulators representing 10 countries (i.e. Armenia, Azerbaijan, Belarus, Georgia, Kazakhstan, Kyrgyzstan, the Republic of Moldova, the Russian Federation, Tajikistan and Ukraine) participated in the workshop for Russian speaking countries in European Region [11]. All participants including experts from manufacturers’ associations joined the case study exercise at the WHO workshops.

The quality attributes presented in this case study do not cover all representative quality attributes of EPO for biosimilarity assessment but a subset to highlight the importance of evaluation of certain quality attributes in relation with their role and influence on clinical performance as outlined in WHO guidelines [1]. The information and data in this case study are fictitious and do not represent products approved or under development. However, the case is designed based on real-life examples of analytical studies for epoetin products and are suitable to highlight important aspects in the evaluation for biosimilarity. The data presented in this case study are simplified graphical data based on real scientific observations. The following questions are addressed to navigate to the better understanding of the principles concerned.

**1**^st^ **question:** Do the quality data for the SBP candidate EPO product (SBP1) demonstrate adequate biosimilarity with the reference product?

a. Potential impact on efficacy
  i. Do the observed differences in post-translational modifications have potential impact to in vivo potency and pharmacokinetics? Is it possible to predict the impact of two offsetting differences in glycosylation?
  ii. Are the non-clinical in vitro and in vivo studies sufficient to conclude that there would be similar potency in patients? Could additional non-clinical studies inform this question?
b. Potential impact on safety/immunogenicity
  i. Do the observed differences in microscopic (subvisible) particle levels have potential impact on immunogenicity? What additional analytical characterization studies might inform this question?
  ii. Are the non-clinical rodent studies adequate to show comparable immunogenicity?

**2**^nd^ **question:** How could “residual uncertainty” be addressed in the clinical studies?

a. Impact of potency
  i. Would a greater than 20% difference in potency be relevant to similarity of efficacy for a therapy that is titrated at the individual patient level?
  ii. If so, how could clinical comparability studies exclude the possibility of a 20% potency difference?
b. Impact of immunogenicity

Low-titer (i.e. clinically benign) anti-EPO binding antibodies may be detected with about 1% incidence in patients treated with epoetins. High-titer, neutralizing antibodies are very rare, and PRCA has an incidence of <1 per 10,000 patient-years.

i. Given these observations, what questions about similarity with respect to immunogenicity can be answered in a reasonably sized pre-licensing clinical study (i.e. with 100–300 patients treated with the candidate biosimilar)?
ii. Can such a pre-licensing study rule out a 10-fold increased risk of PRCA relative to background?
iii. How could the risk of PRCA be addressed in a post-marketing risk management plan?

## 5 Interpretation of the case study

### 5.1 Limitations

Interpretation of the case study is limited to the data and information presented in section 3 above and in Fig. 1. For practical reasons and a short time for the case study, a limited package of quality data and substantially more would be required for a rigorous assessment. No clinical data is presented, but biosimilar assessment requires some clinical data, and this is especially the case for EPO biosimilars. In view of this, the interpretation also includes an assessment of whether the quality data presented for SBP1 is sufficient to allow it to progress to the clinical trial stage.

### 5.2 Overall interpretation

Overall, the data presented in section 3.3 suggests that SBP1 is substantially similar to the RBP. The amino acid sequences are identical (an essential requirement for biosimilars) and the higher order structure of the two products seems equivalent when the limitations of the methods used are taken into account. However, assessment of secondary and tertiary structure of EPO is not easy and a full assessment of this would require further study.

The potency data also supports biosimilarity. Receptor binding seems very similar for the two products and the in vivo bioassay data is also similar; however, the variation in the methodology cannot exclude relatively small differences in potency (e.g. approximately 20%). It is possible that such differences could influence in vivo responses in patients, and ultimately, the required therapeutic dose for maintenance of haemoglobin.

The rat toxicokinetic study demonstrates that SBP1 and the RBP seem similar in their ability to increase haemoglobin levels in animals. However, this data is limited by intrinsic problems with the animal model, which includes induction of neutralizing antibodies in animals after repeat dosing which inhibits the effects of administered EPO.

The information included in Fig. 1 is less reassuring concerning biosimilarity and some of this may impact on the suitability of SBP1 as a biosimilar of the RBP.

The O-linked glycan content of SBP1 shows slightly higher sialyation compared to the RBP, whereas the N-linked sialyation of SBP1 is lower than for the RBP. But the N-linked neutral lactosamine (lactosamine repeat) range is higher for SBP1 than for the RBP. Lactosamine repeats are disaccharide motifs that increase the mass and bulk of sialylated N-glycans without impacting their net charge. The neutral lactosamine content influences clearance in a positive manner. It is therefore difficult to predict the overall effect of the differences noted in sialylation/lactosamine content as these may be to some extent offset by each other, i.e. the higher O-linked sialylation and lactosamine content of SBP1 compared to the RBP may be compensated for by the lower N-linked sialylation of SBP1, producing an overall similar in vivo potency in patients.

Size exclusion HPLC shows a lower level of soluble aggregates for SBP1 compared to the RBP and levels of higher order aggregates are shown to be almost identical for the two products using this technique. This is re-assuring for the demonstration of similarity in terms of immunogenicity of the biosimilar. However, light obscuration reveals higher levels of insoluble aggregates for SBP1 compared to the RBP and this may suggest increased immunogenicity.

The levels of hematopoietic cell phosphatase (HCP) are lower for SBP1 than for the RBP as is the level of N-glycolylneuraminic acid (NGNA), which might imply lower immunogenicity. Overall this suggests that immunogenicity needs to be assessed directly in patients to ensure that SBP1 does not show enhanced immunogenicity compared to the RBP. As immunogenicity is a known problem for EPO (although its incidence in patients is low) and consequences (PRCA) are serious, a careful clinical study is necessary to resolve this issue. The rat toxicokinetic study provides no useful immunogenicity data as human sequence EPO is strongly immunogenic in all rodents unlike in humans and so is of no predictive value.

In conclusion, SBP1 requires further investigation to clarify potential potency and immunogenicity issues. The production process for the product should also be modified to reduce levels of insoluble aggregates and HCPs. Some further investigation may be possible in vitro, but an appropriate clinical trial is clearly required to confirm the biosimilarity of SBP1 compared to the RBP. This should be conducted with the final version of the product and be of appropriate power to resolve the above-mentioned issues.

### 5.3 Responses to the questions posed

The responses from participants of WHO implementation workshops are summarised.

**1**^st^ **question:** Do the quality data for the SBP candidate EPO (SBP1) demonstrate adequate biosimilarity with the reference product?

*Response: As stated in*section 5.2*above, the limited data support biosimilarity of SBP1 and the RBP. However, this needs confirmation by (possibly) further* in vitro *data and (definitely) a clinical trial.*

a. Potential impact on efficacy
  i. Do the observed differences in post-translational modifications have potential impact to in vivo potency and pharmacokinetics? Is it possible to predict the impact of two offsetting differences in glycosylation?
  ii. Are the non-clinical in vitro and in vivo studies sufficient to conclude that there would be similar potency in patients? Could additional non-clinical studies inform this question?

*Response: The impact of the offsetting differences may be to produce an overall similar* in vivo *potency in patients. But this must be confirmed in an appropriate clinical trial. The non-clinical* in vitro *and* in vivo *studies presented are not entirely sufficient to conclude that there would be similar potency in patients. This must be confirmed in an appropriate clinical trial. Further non-clinical studies are unlikely to provide much more valuable information (other than enlarging the data sets).*

b. Potential impact on safety/immunogenicity
  i. Do the observed differences in microscopic (subvisible) particle levels have potential impact on immunogenicity? What additional analytical characterization studies might inform this question?
  ii. Are the non-clinical rodent studies adequate to show comparable immunogenicity?

*Response: The higher levels of insoluble aggregates for SBP1 compared to the RBP shown by light obscuration is a concern for immunogenicity, but this depends on the nature of the particles detected. If they are proteinaceous then their nature and size may cause them to induce immunogenicity in patients. However, if they are non-proteinaceous, e.g. composed of silicone oil in the form of droplets, then these may be innocuous from the immunogenicity perspective. Given the serious nature of immunogenicity for EPO products and the fact that SBP1 is very similar to the RPB which has been associated with immunogenicity issues in the past, this potential problem needs further investigation. Additional* in vitro *analytical studies are possible but limited in sensitivity. Therefore, head to head clinical immunogenicity studies will be necessary to address this. In view of the low incidence of antibody induction in patients treated with EPO, post-marketing surveillance of patients will be required to enable any immunogenicity to be fully assessed. The non-clinical rodent studies are of no use in predicting immunogenicity in patients as all animals will respond to the ‘foreign’ human sequence EPO, whereas this is not the case with human subjects.*

**2**^nd^ **question:** How could “residual uncertainty” be addressed in the clinical studies?

a. Impact of potency
  i. Would a greater than 20% difference in potency be relevant to similarity of efficacy for a therapy that is titrated at the individual patient level?
  ii. If so, how could clinical comparability studies exclude the possibility of a 20% potency difference?

*Response: A greater than 20% difference in potency between SBP1 and the RBP would suggest that there is difference in potency between the two which does not support biosimilarity. This is independent of the dosing regimen used for EPO products. Although this difference may be further investigated by carrying out more bioassays, this is problematic as the primary bioassay is carried out using animals. It should be investigated in a head to head clinical comparability study, with sufficient power and using appropriate endpoints, such as haemoglobin and haematocrit. Such a study should reveal any clinically significant difference in the* in vivo *potency of SBP1 and the RBP.*

b. Impact of immunogenicity

Low-titer (i.e. clinically benign) anti-EPO binding antibodies may be detected with about 1% incidence in patients treated with epoetins. High-titer, neutralizing antibodies are very rare, and PRCA has an incidence of <1 per 10,000 patient-years.

i. Given these observations, what questions about similarity with respect to immunogenicity can be answered in a reasonably sized pre-licensing clinical study (i.e. with 100–300 patients treated with the candidate biosimilar)?
ii. Can such a pre-licensing study rule out a 10-fold increased risk of PRCA relative to background?
iii. How could the risk of PRCA be addressed in a post-marketing risk management plan?

*Response: A pre-licensing clinical immunogenicity study with 100-300 patients treated with SBP1 will reveal any unexpected, exaggerated immunogenicity with a reasonable incidence and such incidence would seriously compromise biosimilarity with the RBP. However, such a trial has too few subjects to validly assess the known serious (but low incidence) immunogenicity associated with PRCA and with the RBP. The study should be able to detect a 10-fold increase in immunogenicity relative to background and this would be a potential indicator that this may be associated with development of PRCA, although confirmation of this would require further clinical study. The risk of PRCA needs to be addressed in post marketing (phase IV) studies conducted on an ongoing basis, e.g. for at least 1 year. This stipulation must be in the risk management plan.*

## 6 Finding and lessons learnt during the case study practices

### 6.1 Assessment for potential impact on efficacy

Differences in glycosylation pattern of the candidate SBP were observed. The groups thought that the observed differences in post-translational modification might or might not have an impact on in vivo potency and pharmacokinetics. However, all groups agreed that regulators would need more quantitative information from the manufacturer to justify the differences.

The biological activity was measured by relevant biological assays. In vitro assays measured cell proliferation; and in vivo assays measured the release of red blood cell precursors into the blood. The groups noted that the nonclinical data (i.e. equivalent potency in vitro proliferation bioassay and in vivo mouse bioassays; and similar rate of increase in haemoglobin levels in rodents) look similar but the limitation of nonclinical studies should be taken into consideration. All groups considered that nonclinical in vitro and in vivo studies alone may not be sufficient to predict the potency in patients. In addition, study data revealed the presence of anti-drug antibody formation in mice after exposure to the candidate SBP. Thus, comparability clinical data are probably necessary to establish similar efficacy.

### 6.2 Assessment for potential impact on safety/immunogenicity

All groups thought that the observed differences in microscopic particle levels could have potential impact on immunogenicity. The light obscuration method used for detecting the higher levels of subvisible particles in the candidate SBP was considered the most reliable assay technique, but the groups agreed that the regulators would ask the manufacturer to use different assays to get information about the identity and quantity of the particles and to conduct stability testing to detect degradation products.

However, no group questioned a potential gap in the capability of the manufacturer's selected techniques to detect proteinaceous higher order soluble and insoluble aggregates that may be potentially immunogenic. Measurement of subvisible particle content by light obscuration is quite insensitive to the particle composition and cannot rule out the possibility of higher levels of proteinaceous particles. The other analytical techniques (i.e. chromatographic or physical techniques) used by the manufacturer suggest that the levels of soluble aggregates (<0.1 μm) are comparable, but the sensitivity of these techniques to higher order species (i.e. larger than trimers) is quite limited. The manufacturer might employ additional, orthogonal techniques to attempt to characterize these aggregates and to differentiate proteinaceous particles from other materials such as silicone oil droplets, but these techniques may not be sensitive to differences at the levels present in the samples.

All groups were of the opinion that nonclinical rodent studies (i.e. repeat dose toxicokinetic studies in mice) are inadequate to predict a similar risk of human immunogenicity because a high percentage of mice will immunoconvert to the foreign protein. Therefore, regulators would suggest that the manufacturer performs head-to-head clinical immunogenicity studies in the most sensitive patient subgroup to generate more relevant data on immunogenicity. Such a study may take 9–12 months, since it may take at least nine months for neutralizing antibodies to develop that will cause PRCA in patients.

### 6.3 Assessment of residual uncertainty in clinical studies

All groups noted that a greater than 20% difference in potency may be relevant for the assessment of similarity of efficacy for a therapy that is titrated at the individual patient level. To gain more confidence in the safety of the candidate SBP, the manufacturer should provide more information about the confidence interval for the potency specification compared to the reference product, as well as clinical data with specific endpoints, such as hemoglobin, haematocrit, antibody titers, type of antibodies (neutralizing or not).

Regarding the reasonable sample size of the pre-licensing clinical study to assess the biosimilarity with respect to immunogenicity, the group noted that a pre-licensing clinical study should be able to detect a 10-fold increased risk in the incidence of low-titer anti-epoetin binding antibodies as are detected with about 1% incidence in patients treated with EPO. As PRCA has an incidence of <1 per 10,000 patient-years, such a study is unlikely to detect PRCA. However, the groups found that the study would provide useful information about the risk of immunogenicity in a candidate biosimilar.

A safety study normally requires approximately 300 patients to provide reasonable power to detect adverse events with a 1% or greater incidence. PRCA has a background incidence of less than 1 per 10,000 patient-years with chronic subcutaneous EPO therapy, and therefore it is practically impossible to power a clinical study to detect a small increase in PRCA risk. However, a >100-fold increase in PRCA risk (i.e. approximately 1% or greater incidence) could potentially be detected in a pre-licensing clinical study. All groups considered that the manufacturer should be requested to submit a risk management plan and pharmacovigilance plan to obtain more information about the potential risk of PRCA, since it is not possible to define the safety profile in its entirety in the pre-licensing clinical study.

In accordance with WHO guidelines [1], “safety data obtained from the clinical trials can be expected mainly to detect frequent and short-term adverse events/reactions. Such data are usually sufficient pre-licensing, but further close monitoring of clinical safety of the biosimilar is usually necessary in the post-marketing phase”. In particular, “when clinically meaningful or even serious antibody development has been encountered with the reference product but is too rare to be captured pre-licensing (e.g. cross-reacting neutralizing anti-epoetin antibodies causing PRCA), a specific risk management plan for the biosimilar may be necessary to assess this specific risk post-marketing”.

Two groups considered that the plan should take into consideration monitoring haemoglobin levels, haematocrit, and antibody titres as an alert signal alerting to increasing occurrence of PRCA. Additionally, one group mentioned including a plan to educate the physician on monitoring of patients. At least one group thought that an observed safety risk of the product could be addressed by the regulators via a variety of regulatory actions that may include product withdrawal as the last option. Apart from product withdrawal, other actions were also noted, e.g. the restriction of the use of the product to specific patient populations, the exclusion of very sensitive groups at risk or the prescription by specialized medical doctors or hospitals. Another group emphasized the importance of having a mechanism for tracing the biosimilars when on the market through indicators, e.g. brand name, manufacturer's name, lot number.

Although it was not discussed at the workshop, it is important to emphasize that the duration of pre-licensing safety studies is also critical to characterize the safety profile of the biosimilar in addition to a sufficient number of patients [1]. “The required observation period for immunogenicity testing will depend on the intended duration of therapy and the expected time of antibody development”. For example, six-month comparative data may not be sufficient since it takes, on average, about 12 months for PRCA to develop. For comparative phases shorter than 12 months, the manufacturer will need to explain why this does not increase the uncertainty about the immunogenic potential of the biosimilar epoetin. In other words, the manufacturer may adopt a shorter comparative phase but takes a certain risk with this approach. In any case, overall 12-month immunogenicity data on the test product needs to be provided pre-licensing [12].

## 7 Conclusions

“Like other biotherapeutic products, SBPs require effective regulatory oversight for the management of the potential risks they pose and in order to maximize their benefits [1].” The experience and expertise of national regulatory authorities in evaluating biotherapeutic products are a key prerequisite for appropriate regulatory oversight of these products. However, after practicing this case study in the WHO implementation workshops on regulatory evaluation of biosimilars, challenges were identified by regulators from developing countries [4]. For example, some regulatory authorities have dedicated assessors for biotherapeutics and SBPs and have developed considerable levels of competency in the assessment of such products, but this is not the case in other national authorities. This causes a lack of understanding of the critical issues in assessing the quality, safety, and efficacy of biotherapeutics including SBPs. Consistent technical support from WHO is therefore requested to understand the impact of differences in comparative quality assessment of SBPs and RBPs and determine whether the differences are acceptable or relevant [13]. In addition, regulators need to increase their knowledge of the relationship between product quality attributes and clinical outcomes of products. It is stressed that the evaluation process requires input from reviewers who may be in different units (quality, clinical, pharmacovigilance) so it should be ensured that they have productive discussions before making decisions [4]. Different national regulatory authorities in a Region may be at different levels of competency in biotherapeutic regulation. Lack of expertise requires capacity building which is a lengthy, resource intensive process. Work sharing can be a possible avenue for developing expertise within a Region as a short-term measure, but constant efforts are requested from WHO to provide well-structured training taking account of the level of competencies of regulators as a long-term approach.

## Disclaimer

The authors alone are responsible for the views expressed in this article and they do not necessarily represent the views, decisions or policies of the institutions with which they are affiliated.

## Declaration of competing interest

Gustavo Grampp is employee of Amgen Inc. The other authors have disclosed no potential conflicts of interests.
